# “The sound of silence”: when the brain doesn’t hear

**DOI:** 10.1007/s40520-026-03412-5

**Published:** 2026-06-08

**Authors:** Elisa Martinelli, Pasqualina Sapone, Pietro Gareri, Massimiliano Massaia, Anna Abbaldo, Angelo Di Stefano, Filomena Padulo, Laura Schiara, Rosaria Carlucci, Enrico Maria Cotroneo, Ilaria Gareri, Antonino Maria Cotroneo

**Affiliations:** 1 Complex Geriatric Unit Maria Vittoria Hospital, Turin, Italy; 2 Resident in training, School of Geriatrics, University Magna Graecia of Catanzaro , Catanzaro, Italy; 3 Geriatrician CDCD Catanzaro Lido, ASP Catanzaro, Catanzaro, Italy; 4 University of Turin, Turin, Italy; 5 Psychologist, Turin, Italy; 6 Complex Geriatric Unit Maria Vittoria Hospital , Turin, Italy

**Keywords:** Older patients, Hypoacusia, Cognitive impairment, Age-related hearing loss

## Abstract

Cognitive impairment is one of the leading causes of disability worldwide and it affects about 6.5% of the population over the age of 65. Hearing impairment is a significant health problem and a possible cause for dementia. Preventing hearing loss or at least correcting it by the use of acoustic prosthesis may reduce the onset of dementia in old age. Indeed, hearing loss has been proposed as a risk factor for dementia, even if the mechanisms that correlate the two diseases are not yet fully understood. It can accelerate existing mild cognitive impairment by increasing the cognitive burden and/or exhausting existing cognitive compensatory strategies, and it could also increase social isolation. Peripheral hearing impairments and Central Auditory Processing (CAP) dysfunction should both be evaluated. Indeed, although peripheral auditory function impairment is perhaps the primary pathological component of early age-related hearing loss (ARHL), CAP dysfunction becomes increasingly important in the later stages of ARHL. The assessment of peripheral hearing function includes self-reporting and some screening tests (such as the 10-item Hearing Handicap Inventory for the Elderly - Short Form), a first-level clinical assessment for hearing loss (such as spoken voice or finger friction tests), audiometric screening tests (portable audiometers, portable audio) and diagnostic audiometric tests (such as pure tone audiometry in a soundproof booth to test the acoustic threshold). The present article focuses on the possible connection between sensory deprivation and the development of cognitive impairment in late age. The use of hearing aids and cognitive training could help delay or prevent the onset of cognitive decline. Furthermore, the use of hearing aids can be an effective tool not only to minimize perceived disability in older individuals, but also to reduce the caregiver’s burden by improving the patients’ ability to communicate.

## Introduction

Cognitive impairment is one of the leading causes of disability worldwide and it affects about 6.5% of the population over the age of 65 [1]. There are currently no treatments that can change the course of the disease, so focusing the attention on risk factors, particularly on modifiable ones (cardiovascular, sensory deprivation - hearing loss), is essential. In fact, data are emerging that point to a decrease in the incidence of dementia probably secondary to social changes and improvements in living conditions, as well as better management of cardiovascular risk [1]. On the other hand, the increase in living expenses of the older population affected by dementia will lead to increased burdens for health services around the world [2].

Since the earliest times, the amplification of sound was considered beneficial in order to connect people, even if the possible consequences of hearing impairment on cognitive functions were not known.

**Fig. 1:**
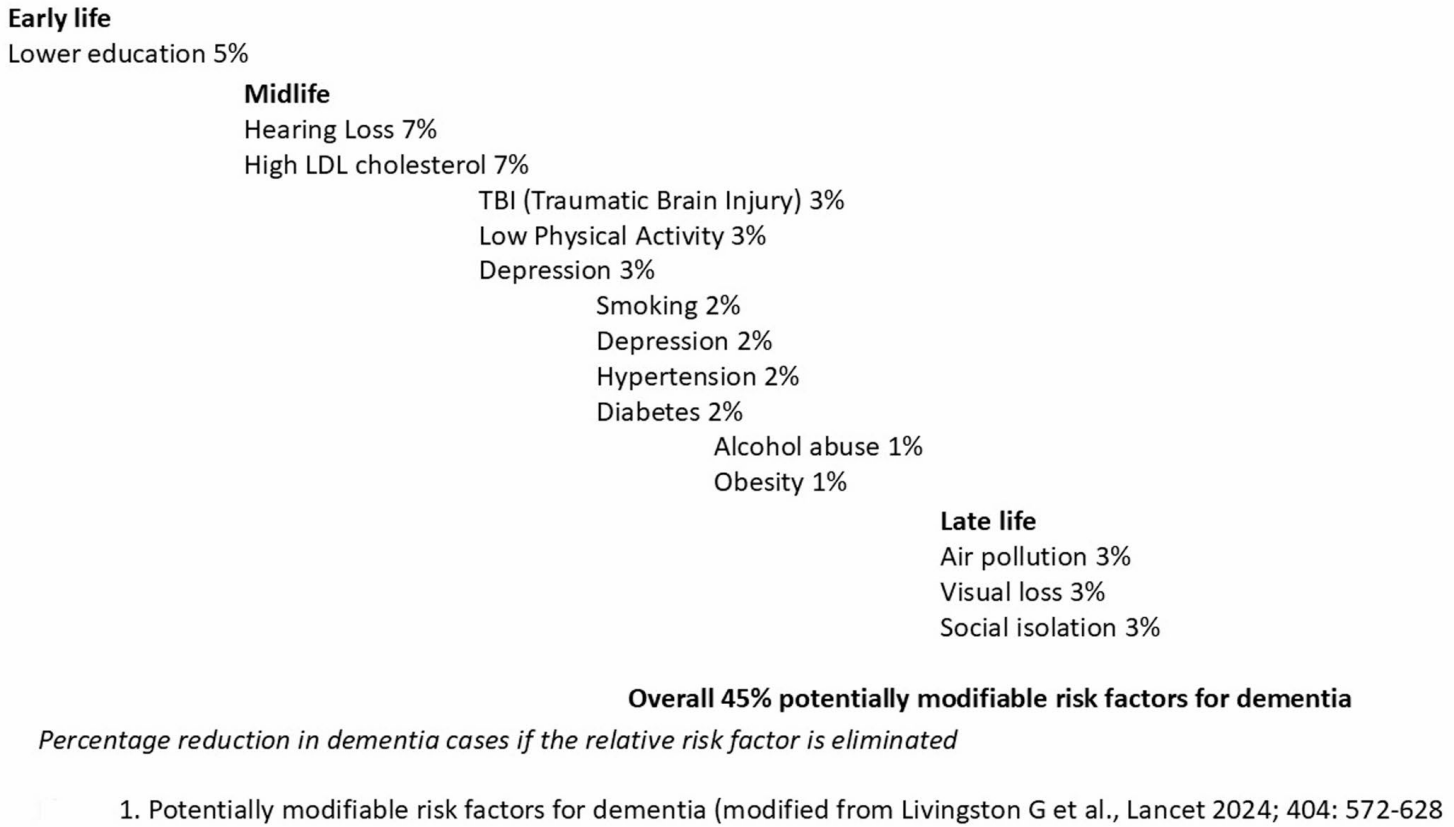
Potentially modifiable risk factors for dementia. (modified from Livingston G et al., Lancet 2024;404:572–6289 [3]).

Figure 1 reports the most important modifiable risk factors for dementia. Among these, hearing impairment is considered crucial for the development of cognitive impairment, but it can be corrected.

Hearing impairment is a significant health problem: the World Health Organization estimates that 5.3% of the world’s population suffers from hearing loss disabilities [2]. From early adulthood, hearing begins to gradually decrease, especially with regard to sounds at higher frequencies. The prevalence of clinically significant hearing loss increases throughout life, almost doubling each decade of life, so that more than two-thirds of all adults aged 60 and over have some form of clinically significant hearing loss [2]. However, half of all cases of hearing loss can be prevented through public health measures [2, 3]. 

The risk of hearing loss increases with age (age-related hearing loss - ARHL) and is estimated to affect up to 40% of those over 65 and up to 75% of those over the age of 80. In Italy there are 7 million people with hearing problems, corresponding to 11.7% of the population. In our country, hearing loss affects one in three people (among those over 65). Only 31% of the population have had a hearing check in the last 5 years, while 54% have never done it. Only 25% of those who could benefit from it use a hearing aid, although 87% of those who use it, declare their quality of life improved [1]. 

### Age-related hearing loss - ARHL

Age-related hearing loss (ARHL) is usually progressive, bilateral and leads to a reduction in one’s ability to communicate. The etiology is often multifactorial with a variety of environmental, medical and genetic determinants. Untreated hearing loss can negatively impact a person’s lifestyle and contribute to social isolation, loss of self-esteem, reduced quality of life and increased risk of neuropsychiatric disorders. The management of ARHL is relatively simple, but hearing aids are expensive and only about a third of those who can benefit from such devices actually buy them, and, even more, a significant percentage of those people do not use them properly [4].

The age-related reduction of hearing abilities gradually affects each individual throughout their life. The ability to hear depends on the inner ear (cochlea) that precisely encodes sounds into neural signals, which are then processed and decoded in meaning at the cortical level. Pathological processes that occur at any level of this pathway, from the ear to the brain, can adversely affect hearing, but age-related hearing loss involving the cochlea is the most common cause [1].

There are several types of age-related hearing impairment, including atrophy of the vascular stria, loss of hair cells and primary degeneration of the cochlear neuron. The specific changes observed in the vascular stria include age-related morphological changes, such as several cytoplasmic vacuoles, enlargement of intracellular spaces, and irregularities of the mitochondria ridges. These changes have been shown to be precipitated by oxidative damage and down-regulation of TMEM16A, a calcium-activated chloride channel. Cochlear disease or trauma can result in hair cell loss, with potential causal factors including continuous exposure to industrial noise, reactive oxygen species (ROS), and Superoxide dismutase (SOD1) deficiency. Primary neural degeneration through the disconnection of auditory neurons from their hairy cell targets during aging has been shown to trigger the loss of hair cells. Studies have identified that the loss of GABA in the central nucleus of the lower collicle and the accumulation of ROS led to neural presbycusis [5].

Age-related hearing loss is characterized by the progressive loss of sensory hair cells in the inner ear, which are responsible for encoding sound into neural signals. Unlike other cells throughout the body, the sensory hair cells in the inner ear cannot regenerate and these cells are progressively lost over the course of life due to the cumulative effects of multiple etiological processes. Risk factors most associated with senility-related hearing loss include advanced age, lighter skin color (as an indicator of cochlear pigmentation - melanin is protective in the cochlea), male sex, and exposure to noise. Other risk factors include cardiovascular risk factors such as diabetes, smoking, and hypertension, which can contribute to microvascular damage to the cochlear blood vessels [6].

In the early stages, ARHL typically affects the audibility of higher frequencies (6,000–8,000 Hz), which interferes with the understanding of normal speech in both quiet and noisy conditions; as the age increases, hearing loss extends to medium and low frequencies. In urban environments, hearing impairment is very common, as intense noise can worsen the deterioration of hearing caused by para-physiological aging. ARHL, usually diagnosed at the stage when speech comprehension is already impaired, is often underdiagnosed at an early stage and consequently, undertreated.

### Age-related hearing loss and cognitive impairment

Hearing loss has recently been proposed as a risk factor for dementia, but the mechanisms that correlate the two diseases are not yet fully clarified. It can accelerate existing mild cognitive impairment by increasing the cognitive burden and/or exhausting existing cognitive compensatory strategies, and it could also increase social isolation by leading to less healthy lifestyle practices, linked to the tendency towards isolation (e.g. smoking, obesity, alcohol abuse), and increased depressive symptoms. It could also induce volume loss in the auditory cortex and other areas of the brain and disrupt the integrity of the white matter’s central auditory tracts and cortical reorganization. In addition, hearing impairment appears to accelerate brain atrophy in the upper, middle, and lower temporal circle and in the hippocampus, areas of the brain commonly implicated in Alzheimer’s disease. Finally, there have been histopathological changes (degeneration, plaques and fibrillar tangles) of the auditory system described in the brain of people with Alzheimer’s disease [7].

Based on these studies, the Lancet Commission on Dementia concluded, in 2020 and in 2024, that hearing loss in middle and late life is one of the main potentially modifiable risk factors for dementia, accounting for 8% of all dementia cases [3]. The main mechanisms through which hearing loss represents a risk factor for cognitive decline and dementia include the negative effects of hearing loss itself and depleted auditory sound coding on cognitive load, brain atrophy and social isolation [3].

### Age-related hearing loss central versus peripheral

In some individuals, hearing problems are determined by Central Auditory Processing (CAP) rather than a peripheral hearing system deficit. Such subjects do not experience problems with speech understanding in a quiet environment, but typically have serious problems in understanding a conversation in a noisy environment, especially when there is simultaneous speech and attentive competition with each other. Peripheral hearing impairments and CAP dysfunction should both be evaluated: although peripheral auditory function impairment is perhaps the primary pathological component of early ARHL, CAP dysfunction becomes increasingly important in the later stages of ARHL [8].

Several procedures are used to diagnose the involvement of the central and peripheral pathways (box 1). The assessment of peripheral hearing function includes self-reporting and some screening tests (such as the 10-item Hearing Handicap Inventory for the Elderly - Short Form), a first-level clinical assessment for hearing loss (such as spoken voice or finger friction tests), audiometric screening tests (portable audiometers, portable audio) and diagnostic audiometric tests (such as pure tone audiometry in a soundproof booth to test the acoustic threshold). CAP measures range from a simple auditory rollover test (with rollover referring to the distortion of words at high signal levels) to complex speech-based behavioral assessments (performing an action in response to a sound signal–synthetic identification sentence with either an ipsilateral competing message [SSI-ICM] or a contralateral competing message; the dichotic digit test [DDT]; or the dichotic sentence identification [DSI] test) (Table 1) [9].

**Table 1:** Tests to assess age-related hearing impairment

Assessment of peripheral auditory function
■ Self-reported hearing impairment■ Clinical screening tests (spoken and/or whispered voice, the sound of a ticking watch, finger friction)■Tonal audiometry■ Screening test (10-item Hearing Handicap Inventory for the Elderly, Short Form [HHIES])
**Assessment of central auditory processing**
■ Auditory rollover test■ Synthetic Sentence Identification with either Ipsilateral Competing Message (SSI-ICM) or Controlateral Competing Message (SSI-CCM)■ Speech Perception In Noise (SPIN)■ Dichotic Digit Test (DDT)■ Dichotic Sentence Identification (DSI) test■ Staggered Spondaic Words (SSW) test

### ARHL and cognitive impairment

The evidence of a possible association between hearing impairment and cognitive impairment, which suggested that hearing loss was more common in patients with dementia, rather than in healthy elderly, was published 50 years ago. Most studies have found an association between ARHL and cognitive impairment [10].

The specific contribution of peripheral and central hearing loss to cognitive disorders in older adults can be difficult to determine, especially in the early stages.

It has also been reported that the correlation between hearing loss and cognitive impairment affects different cognitive domains, such as verbal, episodic and long-term semantic memory, as well as executive function and psychomotor processing [11]. Executive functioning is impaired in patients with Alzheimer’s Dementia, and many of the components involved in CAP, such as short-term memory, attention to task, and inhibition of irrelevant signals, could also result in alterations in executive functioning. In fact, many assessments of central auditory functioning (SSI-ICM, DDT and DSI) and cognitive tests of executive function (Trail-making Tests A and B, Stroop Colour Test, Word Test) explore similar dimensions (e.g., Behavioral Inhibition) of executive control [9].

In the Framingham Heart Study, CAP dysfunction in one ear (assessed with SSI-ICM) was associated with a six-fold increase in the risk of subsequent cognitive decline or development of dementia over the follow-up period of 6 years, and CAP deficit in both ears further doubled the risk [12].

Literature data suggest that CAP deficits could be an early indicator of Mild Cognitive Impairment or Alzheimer’s Disease [13].

Four different mechanisms have been postulated and are reported as follows:

Mechanism 1: the etiopathology common to Alzheimer’s disease (AD) or vascular disease affects the cochlea and/or ascending pathway (causing hearing loss) and the medial temporal lobe (MTL) (causing dementia).

Mechanism 2: the hearing loss leads to a change of the brain structure in the auditory cortex and hippocampus and a decrease in cognitive reserve, and therefore to a decrease in resilience to dementia.

Mechanism 3: the increased brain activity in the MTL and a wider network during speech-in-noise processing competes for the resources needed for other cognition functions.

Mechanism 4: the interaction between altered activity in MTL during difficult listening and AD pathology. There is based the same mechanism for increased activity as mechanism 3. This is based on the interaction between increased activity and synaptic changes associated with AD [13].

We also put forward an alternative mechanism, drawing on new insights into the role of the medial temporal lobe in auditory cognition. In particular, the aberrant activity in the service of auditory pattern analysis, working memory, and object processing may interact with dementia pathology in people with hearing loss. The effects of hearing interventions on dementia depend on the specific mechanisms and suggest avenues for work at the molecular, neuronal, and systems levels [14].

The structural and functional features of auditory brain organization confer vulnerability to neurodegeneration. Moving beyond any simple dichotomy of ear and brain, we argue for a reappraisal of the role of auditory cognitive dysfunction and the critical coupling of brain to peripheral organs of hearing in the dementias (Alzheimer’s disease, Lewy body disease and frontotemporal dementia) [15].

Furthermore, some papers have clearly shown that hearing loss can promote AD via the growth differentiation factor 1 (GDF1) pathway, which interestingly may aid in developing potential AD therapeutic strategies [16, 17].

### Age-related hearing loss, cognitive impairment and frailty

ARHL is also an important indicator of frailty in older adults. Frailty reflects a non-specific state of vulnerability, it is a multisystemic physiological change that predisposes an individual to adverse health outcomes, such as disability, falls, institutionalization, hospitalization and death, and it is of great importance in the older adults clinical care [18].

Psychological, cognitive and social factors all contribute to this multidimensional syndrome. Cognitive impairment is a statistically significant independent factor [19] for adverse outcomes affecting the state of health. Therefore evaluating, managing and preventing the factors that contribute to determining a condition of frailty could be important to prevent adverse events leading to cognitive deficits, such as, for example, delirium and major neurocognitive disorders [13].

Furthermore, it is important to evaluate the relationship between age and the related changes in peripheral auditory function and auditory processing crucial to mitigating the risk of developing cognitive impairment and how frailty could mediate the association between ARHL and cognitive abilities in old age.

The growing body of epidemiological evidence linking ARHL to the cognitive decline of late life suggests various potential mechanisms underlying this association, some of which could be involved in causal pathways that connect ARHL and cognition, while others may constitute common pathological processes or shared etiological pathways that affect both [20].

During the performance of cognitive tasks, the coexistence of hearing disorders negatively affects the performance both in the assessment and rehabilitation, as “degraded” information reaches the patient. Therefore, the correction of hearing impairments with dedicated devices (digital hearing aids, cochlear implants) could directly improve the perceptual processing of speech and therefore improve cognitive functions.

In addition, the overdiagnosis of peripheral ARHL - and therefore, the underdiagnosis of dementia can be a significant problem. However, in subjects with subclinical cognitive impairment, the reliability of audiometric tests seems to be preserved [13]. 

Epidemiological and neuropathological evidence emphasizes how social isolation and loneliness, as a result of communication deficits caused by ARHL, can develop a cognitive decline up to a major neurocognitive disorder [21]. The reduction in cognitive reserve associated with communication deficits was also observed, thus representing a link between ARHL and cognitive impairment. In addition, the cognitive reserve takes into account interindividual differences in susceptibility to age-related brain changes or AD-related pathology, thus modulating the interaction between neuropathology and cognitive impairment [22].

Studies on cognitive reserve with the use of functional magnetic resonance imaging, where participants had moderately reduced peripheral auditory acuity, showed the recruitment of compensatory brain regions affecting the prefrontal and temporo-parietal cortex during the elaboration of a speech. This could explain the general preservation of language comprehension even in the advanced stage of dementia. However, the depletion of the cognitive reserve to help compensate for the hearing and processing deficit could reduce the neural resources available for other cognitive processes, such as working memory and perceptual speed, accelerating and determining the appearance of cognitive deficits [23].

Finally, several factors associated with physical frailty, including inflammatory markers, hormones, nutritional factors, metabolic disorders, and in particular diabetes mellitus, congestive heart failure and stroke, are also linked to cognitive impairment, suggesting that frailty and cognitive impairment share an underlying pathogenesis, probably linked to vascular determinants. Since vascular insults can contribute to both ARHL and cognitive deficits, a shared pathological pathway may exist between ARHL and frailty. In fact, both population studies based on neuropathological data and animal studies have suggested that certain vascular risk factors, such as generalized atherosclerosis, apolipoprotein E variant ε4, could contribute to ARHL [24].

Almost 30 years passed until the recent realization that ARHL is the main preventable risk factor of AD ([25, 26]. Importantly, by removing ARHL there would be a 9.3% reduction of AD cases ([25]. ARHL continues to be overlooked in practice; indeed, it still appears “buried” among other risk factors of AD [7]. Therefore, research efforts converge on efficient treatments of ARHL to reverse or attenuate AD. For example, the cochlear implant is the most successful neural prosthesis available, so that it is essential to optimize outcomes of cochlear implantation in the elderly, as discussed by Illg and Lenarz [27]. They authoritatively highlight factors crucial for such optimization; some crucial points are:

The management of residual low frequency hearing is a priority, and it conditions electrode insertion and electroacoustic simulation;

Comorbidities must be considered;

Pre-existing cognitive decline or impairment call for timely implantation.

Eventually, research will be aimed at identifying additional success predictors and learning curves in the elderly with or without cognitive impairment [28].

In addition, the reported strong association between executive and central auditory function suggests a common mechanism. If the dysfunction of CAP is an indicator of the decline of executive function, it remains largely unexplored at the moment. Other possible mediators of the link between ARHL and cognitive impairment represent a complex clinical phenotype, in which multiple genetic and environmental factors interact: the variation of the glutamate receptor gene that modifies the susceptibility to excitotoxicity of glutamate, as well as the upregulation of the SIRT3 pathway, which regulates the production of superoxide and antioxidants in mitochondria [29].

### Care strategies

One strategy to address age-related hearing loss is to improve a person’s access to speech and other sounds in the hearing environment (for example, music and audible alerts) to promote effective communication, engagement with daily activities and safety. At the moment, there are no repair therapies for age-related hearing loss and condition management focuses on hearing protection, adopting communication strategies to optimize the quality of the incoming auditory signal (on concurrent background noise), and the use of hearing technologies such as hearing aids and cochlear implants.

Communication strategies include encouraging people to be face-to-face and at an appropriate distance when they converse to improve understanding and reduce background noise. Face-to-face communication allows both a clearer auditory signal and the listener to have visual access to facial expressions and lip movements that can help in the central decoding of the voice signal [30].

Hearing aids remain the primary treatment option for age-related hearing loss: they amplify sound and more advanced ones can also increase the signal-to-noise ratio of the desired target sound (for example, by amplifying a speaker’s voice over background noise) by means of directional microphones and digital signal processing, which is critical to improving communication in noisy environments [31].

A Cochrane systematic review concluded that hearing aids in adults improve both hearing-specific outcomes and overall health, and quality of life [32].

People who have more severe hearing loss and who continue to have difficulty understanding speech despite using hearing aids may be candidates for a cochlear implant. The cochlear implant is a neuroprosthetic device that encodes sounds and directly stimulates the cochlear nerve. It is implanted by an otolaryngologist during a surgery that lasts about 2 h. It takes a period of 6 to 12 months after implantation to get used to hearing and perceiving neuroelectric stimuli such as significant speech and sound. Although there is a variance in hearing results after a cochlear implant, improved speech and communication comprehension is often described as “life-changing” by many adults who had long struggled to communicate properly [15].

Another possible approach derives from the study of molecular targets and pathways, because early auditory screening and tailored intervention strategies are crucial for multi-target approaches in managing cognitive decline. Apoptosis, oxidative stress, inflammation, and metabolic dysregulation could be interesting targets to take into account. Indeed, a total of 349 articles published between 2004 and 2024 were retrieved from the Web of Science Core Collection and analyzed using VOS viewer and Cite Space [33]. To this purpose, in the last years there was a steady growth in publication volume, with the United States and China as leading contributors. Molecular analysis identified 2747 genes potentially shared between AD and HL. The study further distinguished between central hearing loss, closely linked to neuroinflammation and synaptic degeneration, and peripheral hearing loss, which potentially contribute to cognitive decline through sensory deprivation [34].

Some of them were shown to be as representative targets related to auditory and neurodegenerative processes (i.e. SLC26A4, CDH23, MYO3A, TMC1, and MYO15A). This is remarkable, because it could be the door for future care strategies.

## Conclusions

Finally, we tried to report a pathophysiological summary of hearing impairment and dementia. Hearing loss may be considered a potential causal risk factor for cognitive decline (risk), a proximity marker for incipient dementia (proximity) or a characteristic of dementia syndrome (phenotype), depending on the time window in which it occurs [34]. The mechanisms of these effects are probably interdependent. In other words, Alzheimer’s disease has been the main target of epidemiological studies that evaluate the risk of developing dementia in association with hearing loss. Hearing loss can account for 10% of all cases of dementia and it has been proposed that it can directly potentiate the effect on the evolution of neurodegeneration. The mechanism suggested by animal models show that it could occur through various mechanism. For example:

- cellular effects such as oxidative stress or altered gene expression;
- changes in neural circuit function;
- a complex interaction between aberrant circuit activity and protein spread.

Until now, a direct causal effect has not been established: for example, peripheral auditory function was not associated with the deposition of cerebral amyloid in a large cohort of cognitively healthy older adults and this effect would not yet represent the majority of cases of dementia with hearing impairments [34]. Therefore, alterations in “central” hearing or auditory cognition may constitute an early warning signal of incipient dementia.

Some authors emphasized that peripheral and central hearing deficits and more general cognitive functions are likely to interact strongly, creating a “vicious cycle” [15].

In particular, in some cross-sectional studies central hearing impairments provide for cerebrospinal fluid tau levels and regional atrophy patterns (for example medial temporal lobe atrophy) consistent with AD and/or the longitudinal development of a clinical syndrome compatible with AD. On the other hand, extensive genetic and neuropathological studies suggested that hearing alterations, in particular, speech perception in noise, may be a preclinical marker of neurodegeneration.

In conclusion, in recent years, increasing attention has been paid to the possible connection between sensory deprivation and the development of cognitive impairment in late life, as such deficits have been associated with an acceleration of cognitive decline. The corrective devices for such deficits (i.e., hearing aids, etc.) and cognitive training could help delay or prevent the onset of cognitive decline and consequently alleviate the fragility and the risk of developing comorbidities and disabilities.

Some randomized controlled trials (RCT) have shown improvement in cognitive function in hearing aid users without cognitive impairment, or with dementia, suggesting that hearing-restoring interventions could be effective in alleviating late cognitive impairment. In addition, the use of hearing aids can be an effective tool not only to minimize perceived disability in older individuals, but also to reduce the caregiver’s care burden by improving patients’ ability to communicate. ARHL is often included in the operational definitions of frailty and cognitive impairment and often represents the risk of a direct and specific association which, if properly treated and managed however, can be reduced [34].

## Data Availability

No datasets were generated or analysed during the current study.

## References

[1] Lin FR (2024) Age-Related Hearing Loss. N Engl J Med 390:1505–1512 38657246 10.1056/NEJMcp2306778

[2] World report on hearing. Geneva: World Health Organization (2021) (https://www.who.intpublications/i/item/ 9789240020481)

[3] Livingston G, Huntley J, Liu KY et al (2024) Dementia prevention, intervention, and care: 2024 report of the Lancet Commission. Lancet 404:572–628 39096926 10.1016/S0140-6736(24)01296-0

[4] Wayne RV, Johnsrude IS (2015) A review of causal mechanisms underlying the link between age-related hearing loss and cognitive decline. Ageing Res Rev 23:154–166 26123097 10.1016/j.arr.2015.06.002

[5] Azeem A, Julleekeea A, Knight B et al Hearing loss and its link to cognitive impairment and dementia. Front Dement 2023. 2. 10.3389/frdem.2023.1199319 10.3389/frdem.2023.1199319 PMC11285555 39081997

[6] Ying G, Zhao G, Xu X et al (2023) Association of age-related hearing loss with cognitive impairment and dementia: an umbrella review. Front Aging Neurosci 15:1241224. 10.3389/fnagi.2023.1241224 37790283 10.3389/fnagi.2023.1241224 PMC10543744

[7] Lin FR, Albert M (2014) Hearing loss and dementia — who is listening? Aging Ment Health 18:671–673 24875093 10.1080/13607863.2014.915924 PMC4075051

[8] Gates GA, Mills JH (2005) Presbycusis Lancet 366:1111–1120 16182900 10.1016/S0140-6736(05)67423-5

[9] Pickles JO (2008) An introduction to the physiology of hearing. Emerald Group Publishing, Bingley, United King- dom

[10] Herbst KG, Humphrey C (1980) Hearing impairment and mental state in the elderly living at home. BMJ 281:903–905 7427503 10.1136/bmj.281.6245.903 PMC1714179

[11] Lin FR (2011) Hearing loss and cognition among older adults in the United States. J Gerontol ABiol Sci Med Sci 66:1131–1136 10.1093/gerona/glr115 PMC3172566 21768501

[12] Gates GA, Anderson ML, McCurry SM et al (2011) Central auditory dysfunction as a harbinger of Alzheimer dementia. Arch Otolaryngol Head Neck Surg 137:390–395 21502479 10.1001/archoto.2011.28 PMC3170925

[13] Füllgrabe C (2020) When hearing loss masquerades as cognitive decline. J Neurol Neurosurg Psychiatry 91(12):1248. 10.1136/jnnp-2020-324707 32855295 10.1136/jnnp-2020-324707

[14] Griffiths TD, Lad M, Kumar S et al (2020) How Can Hear Loss Cause Dementia? Neuron 108(3):401–412. 10.1016/j.neuron.2020.08.003 10.1016/j.neuron.2020.08.003 PMC7664986 32871106

[15] Johnson JCS, Marshall CR, Weil RS et al (2021) Hearing and dementia: from ears to brain. Brain 144(2):391–401. 10.1093/brain/awaa429 33351095 10.1093/brain/awaa429 PMC7940169

[16] Zhao HB, Yang Y (2024) Hearing loss promotes Alzheimer’s disease. Nat Aging 4(4):443–444. 10.1038/s43587-024-00606-2 38491290 10.1038/s43587-024-00606-2 PMC12019645

[17] Chern A, Golub JS (2019) Age-related Hearing Loss and Dementia. Alzheimer Dis Assoc Disord 10.1097/WAD.0000000000000325 PMC7749722 31335455

[18] Gobbens RJ, Luijkx KG, Wijnen-Sponselee MT et al (2012) In search of an integral conceptual definition of frailty: opinions of experts. J Am Med Dir Assoc 11:338–343 10.1016/j.jamda.2009.09.015 20511101

[19] Robertson DA, Savva GM, Kenny RA (2013) Frailty and cognitive impairment—a review of the evidence and causal mechanisms. Ageing Res Rev 12:840–851 23831959 10.1016/j.arr.2013.06.004

[20] Panza F, Solfrizzi V, Logroscino G (2015) Age-related hearing impairment—a risk factor and frailty marker for dementia and AD. Nat Rev Neurol 11:166–175 25686757 10.1038/nrneurol.2015.12

[21] Mick P, Kawachi I, Lin FR (2014) The association between hearing loss and social isolation in older adults. Otolaryngol Head Neck Surg 150:378–384 24384545 10.1177/0194599813518021

[22] Stern Y (2012) Cognitive reserve in ageing and Alzheimer’s disease. Lancet Neurol 11:1006–1012 23079557 10.1016/S1474-4422(12)70191-6 PMC3507991

[23] Boyle PA, Wilson RS, Schneider JA et al (2008) Processing resources reduce the effect of Alzheimer pathology on other cognitive systems. Neurology 70:1534–1542 18354077 10.1212/01.wnl.0000304345.14212.38 PMC10382255

[24] Helzner EP, Patel AS, Pratt S et al (2011) Hearing sensitivity in older adults: associations with cardiovascular risk factors in the health, aging and body composition study. J Am Geriatr Soc 59:972–979 21649629 10.1111/j.1532-5415.2011.03444.x PMC3268119

[25] Montero-Odasso M, Ismail Z, Livingston G (2020) One third of dementia cases can be prevented within the next 25 years by tackling risk factors. The case for and against. Alzheimers Res Ther 12(1):81. 10.1186/s13195-020-00646-x 32641088 10.1186/s13195-020-00646-x PMC7346354

[26] Loughrey DG, Kelly ME, Kelley GA et al (2018) Association of age- related hearing loss with cognitive function, cognitive impairment, and dementia: a systematic review and meta-analysis. JAMA Otolaryngol Head Neck Surg 144:115–126. 10.3389/fnagi.2023.1241224 29222544 10.1001/jamaoto.2017.2513 PMC5824986

[27] Illg A, Lenarz T (2022) Cochlear Implantation in Hearing-Impaired Elderly: Clinical Challenges and Opportunities to Optimize Outcome. Front Neurosci 16:887719. 10.3389/fnins.2022.887719 35903809 10.3389/fnins.2022.887719 PMC9315238

[28] Juiz JM, Fuentes Santamaría V, Scheper V; et al (2023) Editorial: Deafness, aging and Alzheimer’s disease: Neurobiological links and therapy options. Front Neurosci 16:1114383. 10.3389/fnins.2022.1114383 36685247 10.3389/fnins.2022.1114383 PMC9846124

[29] Someya S, Yu W, Hallows WC et al (2010) Sirt3 mediates reduction of oxidative damage and prevention of age-related hearing loss under caloric restriction. Cell 143:802–812 21094524 10.1016/j.cell.2010.10.002 PMC3018849

[30] Schilder AGM, Su MP, Blackshaw H et al (2019) Hearing protection, restoration, and regeneration: an overview of emerging therapeutics for inner ear and central hearing disorders. Otol Neurotol 40:559–570 31083073 10.1097/MAO.0000000000002194

[31] Buchman CA, Gifford RH, Haynes DS et al (2020) Unilateral cochlear implants for severe, profound, or moderate sloping to profound bilateral sensorineural hearing loss: a systematic review and consensus statements. JAMA Otolaryngol Head Neck Surg 146:942–953 32857157 10.1001/jamaoto.2020.0998

[32] Ferguson MA, Kitterick PT, Chong LY et al (2017) Hearing aids for mild to moderate hearing loss in adults. Cochrane Database Syst Rev 9:CD012023 28944461 10.1002/14651858.CD012023.pub2 PMC6483809

[33] Li FF, Wang JP, Zhang WJ et al (2025) Trends and mechanisms of Alzheimer’s disease and hearing impairment: A 20-year perspective. Ageing Res Rev 110:102799. 10.1016/j.arr.2025.102799 40499729 10.1016/j.arr.2025.102799

[34] Palmer CV, Adams SW, Durrant JD et al (1998) Managing hearing loss in a patient with Alzheimer disease. J Am Acad Audiol 9:275–284 9733237

